# An Intervention to Increase Availability of Healthy Foods and Beverages in New York City Hospitals: The Healthy Hospital Food Initiative, 2010–2014

**DOI:** 10.5888/pcd13.150541

**Published:** 2016-06-09

**Authors:** Alyssa Moran, Erica M. Krepp, Christine Johnson Curtis, Ashley Lederer

**Affiliations:** Author Affiliations: Erica M. Krepp, Arlington County Government, Arlington, Virginia; Christine Johnson Curtis, Consultant, Huntington Beach, California; Ashley Lederer, Thoughtful Food Nutrition, New York, New York. All the authors were affiliated with the New York City Department of Health and Mental Hygiene, Long Island City, New York, when the study was conducted.

## Abstract

**Background:**

Hospitals serve millions of meals and snacks each year; however, hospital food is often unhealthy. Hospitals are ideal settings for modeling healthy eating, but few programs have sought to improve nutrition in all venues where food is served.

**Community Context:**

The New York City Department of Health and Mental Hygiene created the Healthy Hospital Food Initiative (HHFI) to improve the healthfulness of food served in hospitals. The HHFI built on prior work implementing mandatory nutrition standards for patient meals and vending in public hospitals. Public hospitals joined the HHFI by voluntarily adopting standards for cafeterias and cafés. Private hospitals joined by implementing nutrition standards for patient meals, food and beverage vending machines, and cafeterias and cafés.

**Methods:**

Hospitals were recruited from 2010 through 2014 and provided technical assistance from health department staff. Implementation in each of the 4 areas was monitored through on-site assessments and menu review. Twenty-eight hospital cafeterias and cafés were evaluated at baseline and at the end of the HHFI to assess changes.

**Outcome:**

Sixteen public hospitals and 24 private hospitals joined the HHFI. Most (n = 18) private hospitals implemented standards in at least 2 areas. In cafeterias, most hospitals introduced a healthy value meal (n = 19), removed unhealthy items from the entrance and checkout (n = 18), increased whole grains to at least half of all grains served (n = 17), and reduced calories in pastries and desserts (n = 15).

**Interpretation:**

Most New York City hospitals joined the HHFI and voluntarily adopted rigorous nutrition standards. Partnerships between hospitals and local government are feasible and can lead to significant improvements in hospital food environments.

## Background

In New York City (NYC), overweight and obesity affects 56.1% of adults (1). Nearly one-quarter of New Yorkers report fair or poor dietary habits, 23% of adults drink at least one sugary drink daily, and nearly 90% consume fewer than 5 servings of fruits and vegetables each day (1). Institutions like government agencies, schools, and hospitals serve food to a large number of people and can improve diets by modeling healthful practices (2). NYC government has transformed the institutional food environment by improving the healthfulness of foods served in schools (3), limiting sugary drinks in childcare settings (4), and issuing mandatory standards for foods purchased and served by government agencies, including public hospitals (5).

Hospitals are ideal venues for modeling healthy eating — a healthful food environment reinforces clinical recommendations and complements hospitals’ missions to support community health. Hospitals provide millions of meals and snacks each year through cafeterias, patient meals, and vending machines (6); however, hospital food is often unhealthy (7–10). Studies show that regular-diet patient menus do not meet dietary guidelines and are high in sodium (8,9). Hospital contracts with fast-food establishments can undermine public health messaging (11), and assessments of vending machines and cafeterias indicate that hospitals are “minimally conducive” to healthy eating (10).

Opportunities exist to improve the hospital food environment, but few programs have sought to improve nutrition in all venues where food is served. Although nutrition standards for vending machines have been widely adopted, until recently there were few models for patient meals and no models for hospital cafeterias, which are important sources of food for employees and are venues that can influence consumer perceptions of a healthy diet (12,13).

## Community Context

In 2008, Mayor Michael Bloomberg issued Executive Order 122, which mandated that all NYC agencies, including public hospitals, adopt nutrition standards for foods purchased and served (NYC Food Standards) (14). Over the following 3 years, nutrition standards for food and beverage vending machines were adopted. The NYC Department of Health and Mental Hygiene (health department) oversaw implementation of the NYC Food Standards and provided technical assistance to agencies. On the basis of successful implementation of these standards in 16 public hospitals, the health department explored working with private hospitals. In NYC, the health care sector is the largest private employer (15), and there are approximately 60 hospitals with potential to reach 8.4 million New Yorkers each year through improvements to the food environment (16,17). Thus, 2 private hospitals were recruited as a pilot program. Working with these 2 hospitals allowed for refinement of the program and showed that hospitals were willing to adopt nutrition standards on a voluntary basis. The pilot also led to the development of the Standards for Cafeterias/Cafés (18), which were created to comprehensively address all venues in hospitals where foods and beverages are served.

In 2010, the health department formally created the Healthy Hospital Food Initiative (HHFI) to improve the healthfulness of foods and beverages available in NYC hospitals. Private hospitals joined by committing to implementing 4 sets of standards: Standards for Patient Meals, Standards for Beverage Vending Machines, Standards for Food Vending Machines, and Standards for Cafeterias/Cafés. Public hospitals, which were already required to meet the Standards for Patient Meals, Standards for Beverage Vending Machines, and Standards for Food Vending Machines as part of Executive Order 122, could join the initiative by voluntarily implementing the Standards for Cafeterias/Cafés.

This article describes the process of partnering with hospitals to implement nutrition standards and assesses key outcomes. First, participation in the HHFI and implementation of each of the 4 standards is described. Second, because nutrition standards for cafeterias were newly developed for the HHFI, implementation of each criterion within the Standards for Cafeterias/Cafés was evaluated at baseline and at the end of the HHFI to assess changes made by hospitals in this setting.

## Methods

The standards used in the HHFI include more than 60 criteria that aim to comprehensively improve the hospital food environment by addressing the nutritional quality of food and beverages purchased and served. The Standards for Patient Meals apply to regular-diet menus and include criteria for foods purchased, such as sodium limits for cereals, and guidelines for meals served (eg, ensuring that 5 servings of fruits and vegetables are offered daily). The Standards for Beverage Vending Machines limit sugary drinks and promote water by reducing sugary drinks to no more than 2 slots in each machine, limiting portion sizes of sugary drinks, eliminating sugary drink advertisements, and placing water at eye level. The Standards for Food Vending Machines set requirements for nutrients (eg, calories, sodium) per package, and promote whole foods, such as dried fruit and nuts, over grain-based snacks. The Standards for Cafeterias/Cafés are 20 criteria to limit sodium, sugary drinks, and calorie-dense foods while promoting water, fruits, vegetables, and whole grains, using a range of strategies. For example, at least half of all grains served in entrees and sandwiches must be whole grains, and 75% of all beverages available must be low-calorie (≤25 kcal/8 oz) (18).

Through relationships established during implementation of the NYC Food Standards and in collaboration with the Health and Hospitals Corporation (HHC), which oversees the administration of city hospitals, public hospitals were approached to join the HHFI by agreeing to voluntarily implement the Standards for Cafeterias/Cafés, in addition to the standards already implemented via Executive Order 122. Support from health department leadership and collaborations with local hospital organizations were key strategies for recruiting private hospitals. The health department cultivated a relationship with the Greater New York Hospital Association (GNYHA) and enlisted the health commissioner to speak directly to private hospital leaders at a GNYHA meeting. Separately, after fostering a relationship with union leaders, the health department sent joint letters from the health commissioner and the 1199 SIEU Hospital Workers’ Union to hospital leaders, asking them to join the HHFI.

### Health department technical assistance

The health department offered technical assistance to hospitals, which included provision of implementation guides, promotional materials, and assistance from 2 full-time registered dietitians. The health department created an HHFI toolkit, which included a description of the nutrition standards and implementation guides. Implementation guides provided general tips for implementing the standards, as well as detailed information to assist with implementation of each individual criterion. For example, the implementation guide for beverage vending machines provided an example of a diagram that could be shared with vendors, showing how machines should be stocked (18).

**Figure 1:**
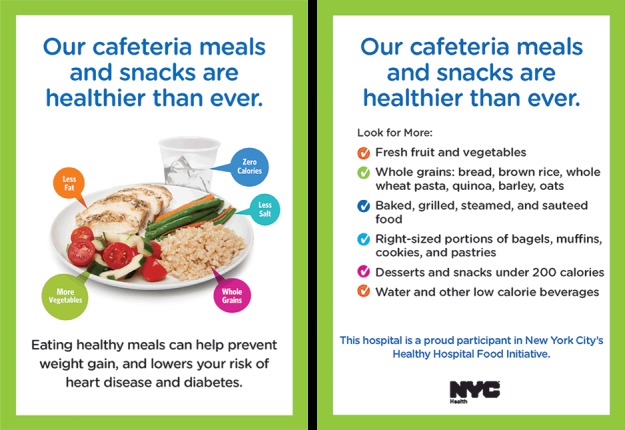
Promotional signage for hospital cafeterias. Abbreviation: NYC, New York City.

The health department encouraged hospitals to communicate with employees about their participation in the HHFI. Hospital communication efforts included employee newsletters, sample food tastings in the cafeteria or café, and cooking demonstrations. The health department created and provided free promotional materials for hospitals to display on vending machines and in cafeterias and cafés, including decals for food and beverage vending machines, promotional signage, table tents (Figure 1), and posters (18).

**Table:** 

The front of the table tent depicts a healthy cafeteria meal. The headline, in large print, reads, “Our cafeteria meals and snacks are healthier than ever.” Balloons that point to parts of the meal describe the improvements. The meat portion is labeled “Less Fat”; a cup of water is tagged “Zero Calories”; vegetables are labeled “Less Salt”; rice is labeled “Whole Grains”; salad is tagged “More Vegetables.” The back of the table tent also reads, “Our cafeteria meals and snacks are healthier than ever.” The text that follows says,
Look for More:
• Fresh fruit and vegetables
• Whole grains: bread, brown rice, whole wheat pasta, quinoa, barley, oats
• Baked, grilled, steamed, and sautéed food
• Right-sized portions of bagels, muffins, cookies, and pastries
• Desserts and snacks under 200 calories
• Water and other low-calorie beverages
• This hospital is a proud participant in New York City’s Healthy Hospital Food Initiative.
The NYC Health logo is at the bottom of the page.

Health department staff, typically a registered dietitian (hereafter, dietitian) and project coordinator, met with the hospital team at each hospital that joined the HHFI to discuss their food service operations, resources, existing wellness initiatives, and goals for improving the food environment. Detailed questions about the hospital’s food service operation and procurement were key to developing a technical assistance plan. For example, health department staff asked whether meals served to patients were planned and prepared by the hospital’s food and nutrition department or contracted through a food service management company, which distributors were used to procure food, and whether the hospital belonged to a group-purchasing organization that negotiates discounts on products with manufacturers and distributors. Health department staff conducted a brief informal assessment of the hospital food environment to help the hospital identify a feasible starting point and together, the team distinguished between standards that could be implemented in the short term and areas that needed work over a longer period. Consistent communication between health department staff and hospital staff was key to keeping hospitals engaged in the HHFI. At a minimum, monthly progress reports were sent to each hospital team, highlighting recent accomplishments and identifying action items and next steps for implementation of each of the 4 standards.

Health department dietitians were available to provide ongoing technical support and, in some cases, worked so closely with hospital food service staff that they became temporary, integrated team members. For example, the dietitian could spend a day in the hospital’s kitchen, taking a detailed product inventory and identifying foods that did not meet nutrition standards. The dietitian had relationships with hospital food service distributors, compiled lists of specific products that met nutrition standards and were available from the hospital’s vendors, and facilitated communication between vendors and hospital staff. The dietitian could also spend several days at the hospital working with the clinical nutrition manager, food service directors, and chefs to update purchasing information and menu analyses.

### Monitoring and evaluation

The number of hospitals joining the HHFI and implementing standards in each of the 4 areas was tracked by using a map of NYC on which each participating hospital’s location was marked with a star. The stars were color-coded according to the hospital’s status in the HHFI; hospitals were awarded a clear star for joining the initiative, a bronze star for implementing nutrition standards in 1 area; a silver star for implementing standards in 2 or 3 areas; and a gold star for implementing standards in all 4 areas. The map was updated approximately every 6 months, circulated to the hospitals, and posted on the health department’s website. The health department also sent certificates to hospital staff members acknowledging their contribution to the HHFI and plaques with a letter from the health commissioner to hospital senior leadership highlighting achievements.

Implementation of the standards was monitored through ongoing conversations with hospital staff, site visits, and menu analyses by health department dietitians. Hospitals were required to meet at least 75% of criteria within each set of standards, with the goal of eventually meeting 100%. To monitor implementation of the Standards for Patient Meals, hospitals submitted regular-diet patient menus and health department staff completed an on-site inventory of purchased food, in which nutrient data were recorded from the Nutrition Facts Labels of all foods stocked in the hospital kitchen and served to patients. For the Standards for Food and Beverage Vending Machines, hospitals were asked to provide a diagram of products stocked in each machine. Health department staff assessed a sample of up to 5 food and 5 beverage vending machines in each hospital, recording the name and package size of each product in the machine, placement of beverages, and presence of advertising. Items were entered into a database, and nutrient data from the manufacturer’s website were used to calculate the nutrient composition of each product. Compliance with the Standards for Cafeterias/Cafés was monitored through site visits and menu analyses by health department dietitians and is described in detail below.

Twenty-eight hospital cafeterias, representing all hospitals that agreed to begin working to improve nutrition in this area, were assessed to evaluate implementation of the Standards for Cafeterias/Cafés during the HHFI. Health department staff assessed cafeterias at baseline, defined as the time the hospital agreed to begin work in the cafeterias or cafés (January 2012–June 2014), and at end line (July 2014–September 2014). Both assessments followed the same protocol. An environmental scan developed for the HHFI was conducted to assess compliance with each of the 20 standards (Table). For criteria requiring nutrient analysis, hospitals submitted monthly menus (for the month of assessment) or cycle menus (if the menu repeated every several weeks), recipes for each menu item, and nutrient data for each product. Health department dietitians conducted nutrient analysis of recipes, favoring nutrient information taken directly from the nutrition facts label of the product purchased by the hospital, when available. To minimize researcher bias, detailed definitions were developed for each standard to ensure consistent assessment and implementation across sites. At each assessment, photographs were taken and checked by a health department staff member to ensure accurate documentation. The number of hospitals implementing each standard was recorded at baseline and end line, and changes were assessed using Pearson’s χ^2^ tests. All analyses were conducted using Stata statistical software, version 13 (StataCorp LP).

**Table:** Number of New York City Hospitals (N = 28) That Implemented Each of 20 NYC Standards for Cafeterias/Cafés, at Baseline Assessment (January 2012–June 2014) and End Line Assessment (July 2014–September 2014), HHFI

NYC Standards for Cafeterias/Cafés	Baseline, n (%)	End Line, n (%)	*P* Value^a^
1. Leafy green salads are available with a vinegar-based dressing option.	25 (89)	28 (100)	.08
2. Four different fruit choices are available.	24 (86)	26 (93)	.39
3. All items contain 0 g trans fat/serving.	22 (79)	26 (93)	.13
4. One vegetable option is available daily (≤200 mg sodium).	18 (64)	26 (93)	.01
5. Water is available at no charge.	14 (50)	24 (86)	.004
6. No use of deep fryers; no deep frying.	6 (21)	9 (32)	.37
7. At least half of desserts are ≤200 kcal/item.	2 (7)	17 (61)	<.001
8. All high-calorie beverages in portions of ≤16 fl oz.	2 (7)	14 (50)	<.001
9. All items are labeled with calorie content.	2 (7)	13 (46)	.001
10. Only healthy options are stocked near the entrance to the cafeteria and at the checkout counter.	1 (4)	19 (68)	<.001
11. Sandwiches are available in half-size portions at no more than half price.	1 (4)	18 (64)	<.001
12. At least half of breakfast pastries are ≤300 kcal/item.	1 (4)	16 (57)	<.001
13. No unhealthy advertising or promotional materials.	1 (4)	16 (57)	<.001
14. All soups are ≤480 mg sodium per 8 fl oz.	1 (4)	12 (43)	<.001
15. At least 75% of beverages are low calorie.^b^	1 (4)	12 (43)	<.001
16. At least half of sandwiches, salads, and entrees are ≤500 kcal; all are ≤700 kcal.	1 (4)	10 (36)	.002
17. Prepackaged snacks meet all nutrient criteria.^c^	1 (4)	9 (32)	.005
18. One healthy value meal is available and priced lower than competing value meals.^d^	0	19 (68)	.001
19. At least half of grain-based sandwiches, salads, and entrees are made with whole grains.	0	17 (61)	<.001
20. At least half of sandwiches, salads, and entrees are ≤800 mg sodium.	0	6 (21)	.01

Abbreviations: NYC, New York City; HHFI, Healthy Hospital Food Initiative; fl oz, fluid ounce.

a *P* values are for Pearson χ^2^ tests comparing proportions of hospitals meeting each criterion at baseline and end line.

b “Low calorie” defined as ≤25 kcal per 8 fl oz.

c Contain per package: ≤200 kcal, ≤7 g fat, ≤2 g saturated fat, ≤200 mg sodium, ≤10 g sugar, ≥2 g fiber if grain- or potato-based.

d Healthy value meal must contain one entrée of ≤600 kcal and ≤800 mg sodium, a fruit or vegetable, and water.

## Outcomes

At the end of the HHFI (September 2014), 16 public hospitals and 24 private hospitals were participating. Participating hospitals represented more than 60% of all acute-care facilities in NYC and by 2014 were located in each borough of NYC (the Bronx, Brooklyn, Manhattan, Staten Island, and Queens). All 16 public hospitals had implemented the standards for patient meals, beverage vending, and food vending before the HHFI, but none had made changes in cafeterias or cafés. By September 2014, 2 (12%) of the 16 public hospitals had implemented the Standards for Cafeterias/Cafés. Although many private hospitals had begun to improve the nutritional profile of foods served before the HHFI, none were implementing the HHFI criteria before joining the initiative. At end line, 17 (71%) private hospitals had implemented the Standards for Patient Meals, 14 (58%) had implemented the Standards for Beverage Vending Machines, 12 (50%) had implemented the Standards for Food Vending Machines, and 16 (67%) had implemented the Standards for Cafeterias/Cafés.

**Figure 2:**
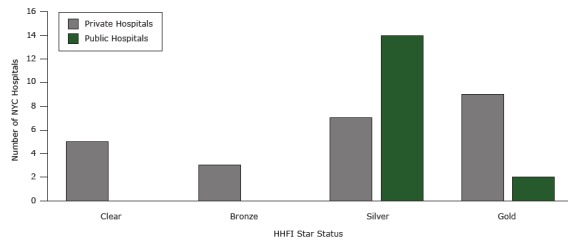
Number of New York City hospitals at each star status at the end of the Healthy Hospital Food initiative (HHFI). The figure represents the number of hospitals participating in the HHFI that achieved each star status as of September 2014. Clear indicates the hospital joined the HHFI but did not implement nutrition standards in any of the 4 areas. Bronze indicates that the hospital implemented nutrition standards in 1 area; silver indicates implementation in 2 or 3 areas; gold indicates implementation in all 4 areas. **Table:** 

The progress of each participating public and private hospital, according to number of standards implemented, is represented by star status (Figure 2). Public hospitals began at silver star status for having already implemented 3 sets of standards, and 2 hospitals reached gold. For private hospitals, 9 (38%) reached gold, 7 (29%) reached silver, 3 (13%) reached bronze, and 5 (21%) joined the HHFI but did not implement standards in any of the 4 areas.

Standard	Private Hospitals	Public Hospitals
Clear	5	0
Bronze	3	0
Silver	7	14
Gold	9	2

Results from the cafeteria evaluation show that hospitals improved the nutritional quality of their offerings (Table). At baseline, most hospitals offered at least 4 different fruit choices (24 [86%]), leafy green salads with a vinegar-based dressing option (25 [89%]), a trans fat–free menu (22 [79%]), and a low-sodium vegetable dish (18 [64%]). No hospitals were meeting the criteria for sodium or whole grains in sandwiches, salads, and entrees, and no hospitals provided a healthy value meal at baseline. At end line, 6 hospitals (21%) met the sodium criterion, 17 (61%) met the criterion for whole grains, and 19 (68%) offered a healthy value meal.

Between baseline and end line, a substantial number of hospitals implemented the breakfast pastry and dessert standards (15 [54%] for each), removed unhealthy foods and beverages from the entrance and checkout of the cafeteria (18 [64%]), offered proportionally priced half-size sandwiches (17 [61%]), offered only soups that met the sodium limit (11 [39%]), and labeled all menu items with calories (11 [39%]). Many hospitals reduced sugary drinks to 25% of all beverages (11 [39%]) and reduced portion sizes of sugary drinks to 16 ounces or smaller (12 [43%]). Fewer hospitals completely removed the deep-fat fryer (3 [11%]), met the criteria for sodium in sandwiches, salads, and entrees (6 [21%]), or met the criteria for pre-packaged snacks (8 [29%]). All hospitals meeting a criterion at baseline were also meeting the criterion at end line.

## Interpretation

Many NYC hospitals joined the HHFI and voluntarily adopted rigorous nutrition standards, which led to significant improvements in hospital cafeterias and cafés. Two-thirds of NYC hospitals joined the HHFI, indicating that the recruitment strategy, which included working with a local hospital association and hospital workers’ union, was effective. Using the standards provided by the health department, hospitals made substantial healthy improvements in their cafeterias, including labeling items with caloric information, reducing availability of sugary drinks, increasing whole grains in sandwiches and entrees, and reducing sodium.

Participation in the HHFI likely benefitted from the context in NYC at the time the initiative was launched. Nutrition policies, such as those requiring city agencies to meet nutrition standards for foods purchased (17), eliminating sugary drinks in childcare settings (4), restricting trans fat in restaurant foods (19), and requiring calorie labels on menu boards in chain restaurants (20), preceded the HHFI. The HHFI was part of a multisectoral effort to improve the healthfulness of foods and beverages citywide and was implemented alongside programs in workplaces, childcare settings, and food retailers. The health department’s work in hospitals was supported by high-level officials, including the health commissioner and HHC leadership, which was integral to connecting with hospital leaders and encouraging participation.

Despite high participation, there were challenges to making changes in hospitals, particularly in the retail environment. First, nearly all vending machines were managed by outside vendors, and early in the HHFI there was limited availability of products that met the nutrient criteria. However, because many hospitals contracted with the same vendor, there was increasing demand to supply products meeting the HHFI criteria as participation in the initiative increased. Second, most hospital cafeterias were operated by each hospital’s food and nutrition department, but several were franchisees of a larger chain, which made it difficult to make local changes when decisions were made at the corporate level. Thus, no hospitals with franchised cafeterias had implemented the Standards for Cafeterias/Cafés at the end of the HHFI. Lastly, few hospitals tracked nutrient content of foods purchased for the cafeteria or had nutrient analysis of the cafeteria menus at baseline, requiring an intensive investment of staff time and technical assistance from health department dietitians.

For the health department, the HHFI supported work to improve the health of New Yorkers by improving the food environment citywide. Additionally, implementing standards in hospitals that are consistent with what is required of city agencies helped to ensure that healthful foods are available in all institutional food service settings, and increased demand for healthful items from food service vendors. For hospitals, the HHFI provided a comprehensive framework for improving the food environment. Hospitals benefited from individualized technical assistance, access to promotional materials, and public recognition for their accomplishments, which are resources that could be leveraged by other local health departments seeking to partner with hospitals on similar initiatives. The HHFI shows that hospitals are willing to partner with local government to voluntarily implement rigorous nutrition standards, which can lead to significant changes in hospital food environments.
